# Synthesis of Riboflavines, Quinoxalinones and Benzodiazepines through Chemoselective Flow Based Hydrogenations

**DOI:** 10.3390/molecules19079736

**Published:** 2014-07-08

**Authors:** Marcus Baumann, Ian R. Baxendale, Christian H. Hornung, Steven V. Ley, Maria Victoria Rojo, Kimberley A. Roper

**Affiliations:** 1 Department of Chemistry, University of Durham, South Road, Durham DH1 3LE, UK; 2 Department of Chemistry, University of Cambridge, Lensfield Road, Cambridge CB2 1EW, UK

**Keywords:** flow chemistry, hydrogenation, riboflavine, benzodiazepine, micro reactor

## Abstract

Robust chemical routes towards valuable bioactive entities such as riboflavines, quinoxalinones and benzodiazepines are described. These make use of modern flow hydrogenation protocols enabling the chemoselective reduction of nitro group containing building blocks in order to rapidly generate the desired amine intermediates in situ. In order to exploit the benefits of continuous processing the individual steps were transformed into a telescoped flow process delivering selected benzodiazepine products on scales of 50 mmol and 120 mmol respectively.

## 1. Introduction

The quest to develop efficient and economic routes towards molecules with potential biomedical applications continues to be one of the most challenging tasks of modern synthetic organic chemistry [1,2,3]. As such medicinal chemistry programs commonly seek to identify valuable heterocyclic core structures that can subsequently be diversified delivering libraries of new compounds for high throughput screenings. While the most recent drug candidates entering clinical trials are likely to be based on new scaffolds it might be surprising to learn that at least two thirds of today’s top-market drugs are based on venerable classical heterocyclic cores clearly indicating the value of such privileged structures [4,5,6].

Even though the search for new promising lead compounds continues to be a constant within pharmaceutical and agrochemical research environments, the last 15 years have witnessed a true step change in how such structures are prepared and evaluated [7,8,9]. This includes the before-mentioned high-throughput screening efforts in order to test compounds rapidly as well as the incorporation of so-called enabling technologies such as microwave and flow reactors [10,11,12,13,14,15,16]. The latter have now gained widespread acceptance in the field as they typically offer improvements in efficiency and safety when accessing valuable chemicals on scales ranging from the initially required milligrams to kilograms and indeed metric ton-scale production [17,18,19,20,21]. Importantly, both industry and academia are driving research in these areas at an impressive rate aiming to improve the often low success rates of current drug development programs [22,23,24,25].

Research in our team over the last decade has focused on the development of new instrumentation [26,27,28,29,30,31] and methodologies [32,33,34] geared towards efficient and innovative flow synthesis. One key area has been the assembly and elaboration of numerous heterocyclic scaffolds and target molecules as required in medicinal chemistry programs [35,36,37,38,39,40]. For example, one of our recent research programs was directed at the synthesis of diversely functionalized pyrrolidines achieved by means of chemoselective hydrogenation of *N*-benzyl-protected 4-nitropyrrolidines which was successfully performed using the H-Cube™ flow system [41,42,43]. This small foot-print flow instrument conveniently and safely performs the electrolysis of water and mixes the *in situ* generated hydrogen gas with the substrate stream. Subsequent passage of the resulting mixture through a heated cartridge containing a heterogeneous catalyst allows for the rapid hydrogenation of various functional groups with residence times typically ranging from 1–5 min. We now wish to disclose our continuation of our earlier findings which further harnesses the many advantages of flow-based synthesis in generating libraries of important bioactives such as riboflavines and benzodiazepins. Riboflavine (vitamin B_2_) is the central component of the redox cofactors flavine adenine dinucleotide (FAD) and flavine mononucleotide (FMN), and its analogues have been reported to display anti-inflammatory, antihyperalgesic, anticancer and antimalarial activities [44,45,46]. The non-natural benzodiazepines on the other hand constitute of a class of molecules possessing psychoactive activity acting on the gamma-aminobutyric acid receptors (GABA receptors) [47,48,49]. Consequently, several of today’s widely prescribed medications such as olanzapine, alprazolam, clonazepam and diazepam contain the benzodiazepine pharmacophore highlighting its continued importance [50,51].

## 2. Results and Discussion

### 2.1. Synthesis of Riboflavins Derivatives

Most commonly, riboflavine analogues can be accessed by the direct condensation of 1,2-diaminobenzene derivatives **2** and alloxane (**3**) [52,53,54,55,56,57]. This transformation renders the tricyclic riboflavines directly if performed in acidic conditions, whereas quinoxalinone derivatives are obtained under neutral conditions. One major drawback of this strategy, however, lies in the limited commercial availability and unstable nature of many 1,2-diaminobenzenes. We therefore elected to use the H-Cube™ flow hydrogenation system in order to prepare the required 1,2-diaminobenzene substrates **2** *in situ* from low cost and easily sourced 2-nitroaniline starting materials **1**. 

**Scheme 1:**
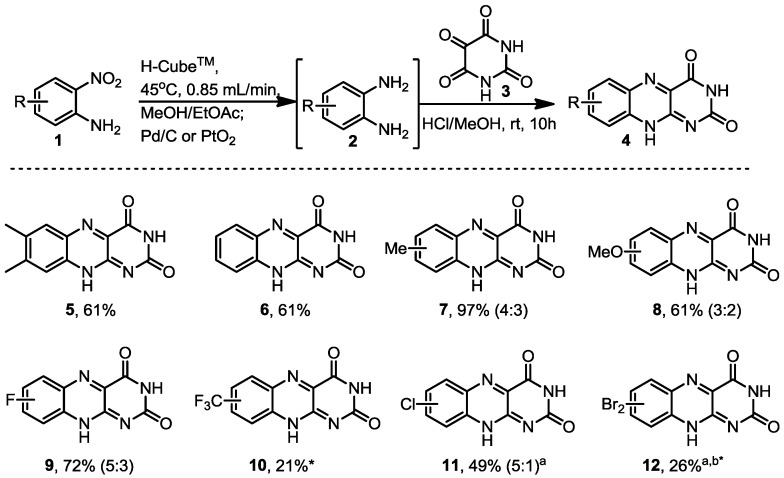
Riboflavine analogues prepared.

^a^ PtO_2_ used instead of Pd/C. ^b^ from 3,5-dibromo-1-nitro-2-aminobenzene. * single regioisomer.

To this end stock solutions of various 2-nitroanilines in methanol/ethyl acetate (1:1 by volume, 0.1–0.25 M) were prepared and pumped into the H-Cube™ system where after mixing with the *in situ* generated hydrogen gas the stream was directed into a pre-packed cartridge containing a heterogeneous metal catalyst. Initial experiments established that the best results were obtained when using a cartridge filled with palladium on carbon (10 wt %). Passing the solution of substrate through this cartridge maintained at slightly elevated temperature (45 °C) delivered a colourless product stream within a residence time of 4–6 min. While this colour change conveniently served as an indication of the complete reduction of the typically yellow 2-nitroaniline starting materials, it was observed that this solution would turn yellow then to brown upon standing, correlating to slow re-oxidation/decomposition of these unstable intermediates. For this reason the 1,2-diaminobenzene intermediates **2** were not isolated, but rather collected in a stirred vial containing alloxane (**3**) (equimolar concentration 0.1–0.25 M) dissolved in methanol containing HCl (5 mol %, rt) to afford the desired riboflavine adducts (**4**). It was found that the final cyclocondensation was best performed in batch at ambient temperature allowing for the isolation of the precipitated products in good yields and excellent purity (Scheme 1).

During our study we found that using a Pd/C catalyst cartridge reliably reduced the 2-nitroaniline inputs, however, in case of substrates containing sensitive chloro- or bromo-substituents concomitant dehalogenation was observed. However, this could be avoided by substituting the Pd/C catalyst for PtO_2_ allowing access to the riboflavine analogues functionalized by chloro and bromo groups which are valuable materials for further elaboration using transition metal coupling chemistries.

**Scheme 2:**
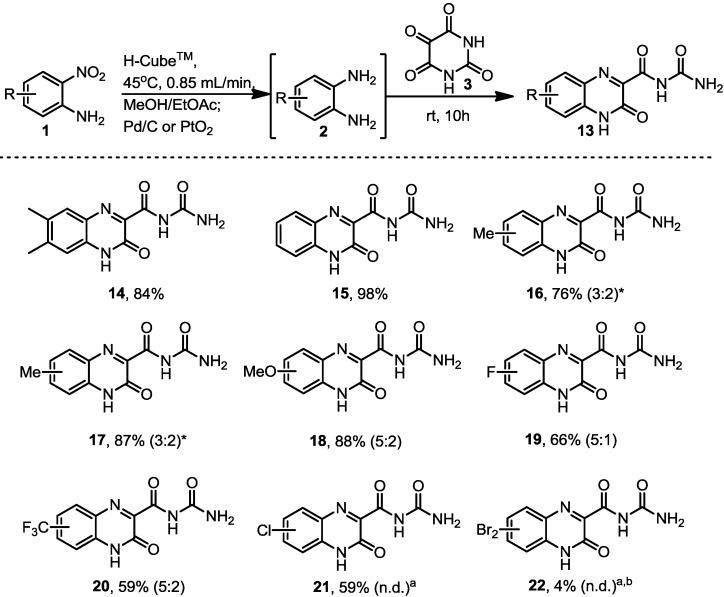
Quinoxalinone analogues prepared.

^a^ PtO2 used instead of Pd/C. ^b^ from 3,5-dibromo-1-nitro-2-aminobenzene. * from regioisomeric substrates.

In addition to this series of riboflavines we also decided to study the synthesis of the related quinoxalinone structures **13** which are obtained when treating the *in situ* generated 1,2-diaminobenzenes with alloxane under neutral conditions. The desired structures were again isolated in good to excellent yields using the H-Cube™ in combination with Pd/C or PtO_2_ as catalysts in the first step (Scheme 2). These materials were extremely polar adducts which were difficult to isolate using standard chromatographic methods, however, a slight modification of the reaction solvent system (3:1 EtOAc/MeOH) furnished the desired products in high isolated yield.

As expected, regioisomeric products were obtained in both series when non-symmetrical starting materials were used. The ratio of the regioisomers was strongly dependent upon the specific substitution pattern mainly arising from electronic differentiation of the 1,2-dinitrogen functionality biasing the initial nucleophilic attack on the alloxane (**3**). As these mixtures could not be separated by column chromatography the combined yield and the corresponding ratio is reported for each pairing. Taking an isolated mixture of compound (**13**) and subjecting it to the acidic conditions used to form the equivalent cyclised structure (**4**) gave a very slow transformation to the desired material (6 days, 45% conversion). Heating the reaction mixture aided solubility and increased the rate of the transformation as did adding polar solvents such as DMSO and DMF. In each case the resulting regioisomeric ratio was directly translated from the starting material (**13**) into the regioisomeric composition of compound (**4**).

### 2.2. Synthesis of Dibenzodiazepine Structures

**Scheme 3:**
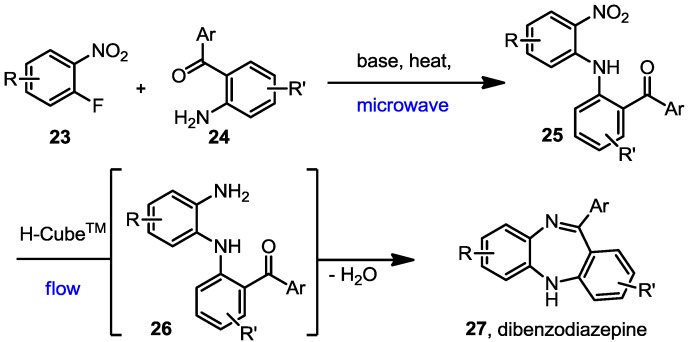
Synthetic route for the synthesis of dibenzodiazepines.

Despite the significant pharmacological potential of various dibenzodiazepine scaffolds very little progress has been made towards developing improved synthetic procedures since the pioneering studies of Schmutz *et al.* in the early 1960s [58,59,60]. In part this might be due to the now well-known side-effects such as long-term tolerance and dependence symptoms of the related benzodiazepine structures [61,62]. However, given the growing demand for medications treating neurological disorders one can expect a renewed interest into these structures, especially if compounds with more desirable long-term profiles can be identified. Furthermore, recent reports have started to reveal the binding mode of benzodiazepines within the GABA_A_ receptor and have consequently stimulated the synthesis and testing of new analogues [63]. Crucial for further advancement will be the availability of robust and efficient methodology delivering new sets of analogues based on this classical scaffold. For this reason we decided to establish a concise route combining a S_N_Ar reaction to first build a nitrodiarylamine intermediate **25**, which upon reduction of the pendant nitro group would furnish an amine, which subsequently undergoes a cyclodehydration reaction affording the desired dibenzodiazepine scaffold (Scheme 3). This process is particular desirable with respect to its atom- and step-economy. 

We initiated our studies by subjecting several 2-aminobenzophenones **24** to S_N_Ar reactions with 2-fluoronitroarenes **23** in the presence of LiHMDS as a base. It was discovered that microwave heating effectively and conveniently generated the desired products **25** in high yields within short reaction times. Following aqueous work-up these diarylamine intermediates were redissolved in acetonitrile or a mixture of CH_2_Cl_2_/MeOH (depending on solubility) and passed through an H-Cube™ flow system operated at slightly elevated temperature (60 °C) in order to accomplish full conversion within ~5 min residence time. Based on the previously observed benefits of using PtO_2_ instead of Pd/C as catalyst within the flow cartridges we decided to use this catalyst throughout this study. Anhydrous MgSO_4_ was placed in-line using a packed glass Omnifit column to assist in the final cyclodehydration process. The desired dibenzodiazepines were typically isolated in high yield following solvent evaporation and chromatographic purification (Table 1).

However, the initial batch microwave S_N_Ar reaction significantly restricted our ability to easily scale up the sequence. This was particularly frustrating as the subsequent flow hydrogenation process was an essentially quantitative transformation completed in a short residence time. Consequently, we wished to establish a more direct and streamlined delivery of material from the S_N_Ar step to create a fully integrated multi-step process.

**Table 1:** Dibenzodiazepine derivatives prepared.

Entry	Dibenzodiazepine Product	Step 1: Time/min	Step 2: Solvent	Yield
1	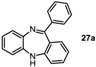	60 ^a,b^	MeCN	65%
2	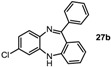	30	MeCN	66%
3	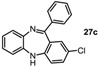	30	CH_2_Cl_2_/MeOH (3:1)	72%
4	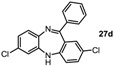	30	MeCN	59%
5	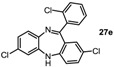	30 ^a^	MeCN	70%
6	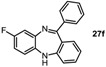	30	MeCN	67%
7	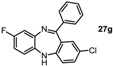	30 ^a^	CH_2_Cl_2_/MeOH (3:1)	58%
8	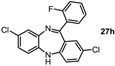	60	MeCN	43%
9	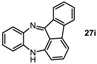	150	CH_2_Cl_2_/MeOH (3:1)	51%
10	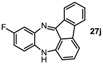	90	CH_2_Cl_2_/MeOH (5:1)	37%
11	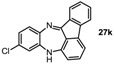	90 ^a^	CH_2_Cl_2_/MeOH (3:1)	48%

^a^ 2 equiv. of nitrobenzene used. ^b^ KHMDS in toluene was used.

**Scheme 4:**
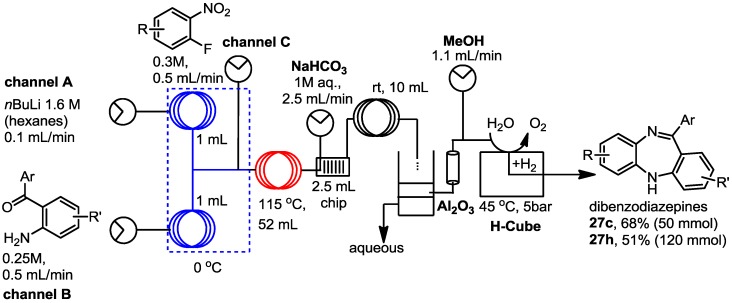
Continuous flow synthesis of dibenzodiazepines.

From initial base screening we had also identified that KO^t^Bu and *n*-BuLi were effective for the S_N_Ar transformation. Therefore, in order to create a potentially continuous process we elected to use stock solutions comprising of 1.6 M *n*-BuLi in hexanes (channel A, Scheme 4) and 0.3 and 0.25 M solutions of 2-fluoronitroarene (**23**; channel C) and 2-aminobenzophenone (**24**; channel B) respectively in 3-methyltetrahydrofuran (3-MeTHF). A simple flow reactor was assembled using two low volume PTFE pre-cooling loops (1 mL, cooled to 0 °C - channels A and B) linked by a T-mixer to a short tube reactor (0.10 mL, rt) in which the rapid deprotonation occurs. A second T-mixer successively unites the third flow stream containing the electrophile (channel C) before the reaction enters a 52 mL heated reactor coil (115 °C) to promote the substitution step. Optimised flow rates of 0.1 mL/min-channel A, 0.5 mL/min-channel B and 0.5 mL/min-channel C, gives corresponding residence times of 10 s and 47.3 min in the two reactor zones. The downstream work-up and purification was performed by the introduction of an aqueous solution of NaHCO_3_ (1 M, 2.5 mL/min) mixed within a 2.5 mL Uniqsis mixer chip and directed into a 10 mL PTFE coil reactor where the mixture developed into plug flow. The resultant biphasic feed was collected into a continuous separation tank (70 mL Biotage Universal Phase Separator-Cat No. 120-1930-V). The aqueous phase was constantly removed using a positioned side arm run off to maintain a fixed liquid volume (~45 mL). The separated organic layer was dried by passage through an exchangeable column of oven dried alumina (120 g) then diluted (1:1) with a MeOH make-up stream prior to entering the H-cube MIDI™ hydrogenator (5 bar, 45 °C, 2.2 mL/min, 116 mm × 9.2 mm, PtO_2_). The reaction step within the H-cube™ was started with a delay of 90 min relative to the first reactor; a buffering reservoir was placed between the two stages to compensate for the volume. Solvent evaporation of the processed solutions allowed isolation of the crude materials (entry 3 and 8 above; **27c** = 50 mmol scale, crude 13.7 g, ~83% purity: **27h** = 120 mmol scale, crude 38.1 g, ~69% purity) in comparable purities and isolated yields (**27c** 68% and **27h** 51%) to the original two-step process. Isolation was achieved by loading the crude material onto samplet cartridges (10 g) followed by chromatography using a Biotage SP4 instrument.

**Scheme 5:**
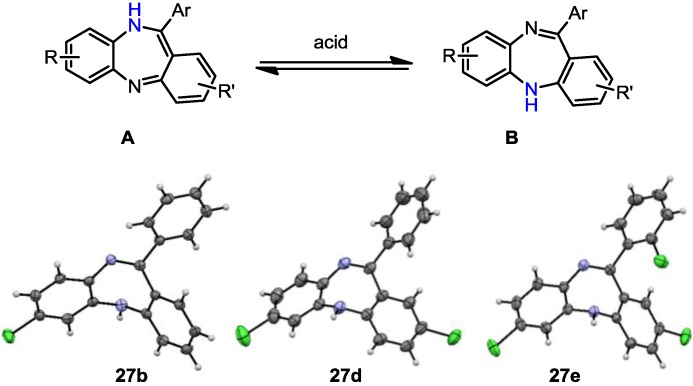
Tautomer interconversion and X-ray crystal structures of compounds **27b**, **27d** and **27e** (nitrogen: blue; chlorine: green).

A particularly notable feature of these dibenzodiazepine structures is the prevalence of different tautomers **A** and **B** that form in a pH-dependent manner (Scheme 5). Based on our observations we conclude that tautomer **A** is the predominant species formed during the cyclodehydration event, which interconverts into tautomer **B** upon exposure to acidic media such as the silica gel used in the purification. In order to prove this assumption, mixtures of both tautomers were dissolved in formic acid (0.15 M, in MeOH/EtOAc, 1:1) leading over the course of 30 min to full conversion into tautomer **B** as evidenced by the results of several single crystal X-ray diffraction experiments. Moreover, these X-ray structures reveal the distinct non-planar shape of these molecules due to the steric impact of the attached aryl substituent resulting in an unusual three-dimensional orientation of these dibenzodiazepine derivatives.

## 3. Experimental

Full experimental details and spectroscopic characterization data is given is the proceeding section: 

### 3.1. General Information

Unless otherwise specified, reagents were obtained from commercial sources and used without further purification. Solvents were obtained from Fisher Scientific and distilled before use. 

^1^H-NMR spectra were recorded on a Bruker Avance DPX-400, DPX-500, or DPX-600 spectrometer with the residual solvent peak as the internal reference (CDCl_3_ = 7.26 ppm, d_6_-DMSO = 2.50 ppm). ^13^C-NMR spectra were recorded on the same spectrometers with the central resonance of the solvent peak as the internal reference (CDCl_3_ = 77.16 ppm, d_6_-DMSO = 39.52 ppm). DEPT135, COSY and HMQC experiments were used to aid structural determination and spectral assignment. 

IR spectra were recorded neat on a PerkinElmer Spectrum One FTIR spectrometer with Universal ATR sampling accessories. Letters in parentheses refer to the relative absorbance of the peak: w = weak (<40% of the most intense peak), m = medium (40%–70% of the most intense peak), s = strong (>70% of the most intense peak).

High resolution mass spectra (HRMS) were recorded on a Waters Micromass LCT Premier Q-TOF spectrometer by electrospray ionisation (ESI) or an ABI/MDS Sciex Q-STAR Pulsar. The mass reported is containing the most abundant isotopes. Limit: ±5 ppm. LC-MS analysis was performed on an Agilent HP 1100 series chromatograph (Mercury Luna 3μ C18 (2) column) attached to a Waters ZQ2000 mass spectrometer with ESCi ionisation source in ESI mode.

### 3.2. General Procedure for the Hydrogenation Reactions in Flow Using the H-Cube^®^

A solution of the nitroaniline (1 mmol, 0.1–0.25 M, MeOH/EtOAc, 1:1) was passed through the H-Cube^®^, which was equipped with a cartridge filled with the corresponding Pd/C or PtO_2_/C catalyst. Extra back pressure regulators were added to the H-Cube^®^ set-up, with a pressure of 100 psi (6.9 bar) being applied before the solution entered the H-Cube^®^ and a pressure of 250 psi (17.2 bar) applied to the exiting solution. The nitroanilines were pumped at different temperatures and flow rates depending on the substrate using full H_2_ mode. The exiting solutions were concentrated to determine the reaction conversion by ^1^H-NMR analysis and used subsequently due to the instability of the diamines. The catalyst cartridge was exchanged approximately every 8 runs. 

### 3.3. General Procedure for the Formation of the Riboflavine Analogues **5**–**12**

The diamines generated by the hydrogenation process in flow were mixed with alloxane monohydrate (0.160 g, 1 mmol) and hydrogen chloride in methanol (1 mL of approx. 1.25 M concentration) and left to stir at room temperature overnight. The product was then filtered and the solid product was dried *in vacuo*.

*7,8-Dimethylbenzo[g]pteridine-2,4(3H,10H)-dione* (**5**). Prepared from 4,5-dimethyl-2-nitroaniline (0.166 g, 1 mmol) to give a yellow solid (0.147 g, 61% yield). ^1^H-NMR (500 MHz, *d*_6_-DMSO): δ/ppm = 11.82 (1H, d, *J =* 1.8 Hz, NH), 11.65 (1H, s, NH), 7.90 (s, 1H), 7.69 (s, 1H), 3.15 (s, 6H, 2 × CH_3_); ^13^C-NMR (125 MHz, *d*_6_-DMSO): δ/ppm = 160.7 (C), 150.1 (C), 146.5 (C), 144.7 (C), 141.7 (C), 139.0 (C), 138.4 (C), 130.3 (C), 128.8 (CH), 125.9 (CH), 55.0 (CH_3_), 48.6 (CH_3_). IR (neat) ν/cm^−1 ^= 3445.6 (w), 3187.7 (w), 3145.6 (w), 3073.2 (w), 2985.0 (w), 2951.7 (w), 2848.1 (w), 1734.3 (m), 1693.0 (s), 1629.8 (w), 1587.9 (m), 1578.0 (m), 1486.7 (w), 1441.5 (w), 1423.8 (w), 1386.7 (w), 1363.8 (m), 1349.3 (m), 1285.7 (s), 1221.3 (w), 1192.6 (w), 1143.9 (w), 1097.6 (w), 1035.0 (m), 1023.5 (w), 1001.9 (w), 911.5 (w), 882.9 (m), 816.2 (m), 797.9 (w), 770.5 (w), 754.5 (m), 736.2 (w), 683.9 (w), 661.9 (w). LC-MS: R_t _= 4.57 min; HRMS (ESI): *m/z* calculated for C_12_H_9_N_4_O_2_ [M+H^+^]: 213.0731; found 241.0732. 

*Benzo[g]pteridine-2,4(3H,10H)-dione* (**6**). Prepared from 2-nitroaniline (0.138 g, 1 mmol) to give a pale green solid (0.131 g, 61% yield). ^1^H-NMR (500 MHz, *d*_6_-DMSO): δ/ppm = 11.94 (d, *J =* 1.6 Hz, 1H, NH), 11.75 (s, 1H, NH), 8.16 (d, *J =* 8.2 Hz, 1H), 7.92 (m, 2H, 2 and 3), 7.77 (m, 1H); ^13^C-NMR (125 MHz, *d*_6_-DMSO): δ/ppm = 160.5 (C), 150.2 (C), 146.9 (C), 142.7 (C), 139.3 (C), 133.4 (CH), 131.8 (C), 130.2 (CH), 128.5 (CH), 127.0 (CH). IR (neat) ν/cm^−1^: 3173.4 (w, br), 3084.4 (w, br), 28.43.1 (w, br), 1813.8 (w), 1786.4 (w), 1733.6 (m), 1687.9 (s), 1618.5 (w), 1582.9 (m), 1505.4 (w), 1485.5 (w), 1447.4 (m), 1390.4 (m), 1364.0 (m), 1334.4 (m), 1316.1 (m), 1271.4 (s), 1248.8 (m), 1212.6 (s), 1153.6 (w), 1144.9 (w), 1034.9 (w), 1015.0 (w), 989.7 (w), 915.6 (w), 866.9 (w), 811.8 (w), 763.6 (s, br), 705.1 (m), 677.8 (m). LC-MS: R_t _1.82 min; HRMS (ESI): *m/z* calculated for C_10_H_5_N_4_O_2_ [M+H^+^]: 213.0418; found 213.0411.

*7-Methylbenzo[g]pteridine-2,4(3H,10H)-dione and 8-methylbenzo[g]pteridine-2,4(3H,10H)-dione* (**7**). Prepared from 4-methyl-2-nitroaniline (0.152 g, 1 mmol) to give a yellow solid, with two regioisomers found in a 4:3 ratio (0.212 g, 96% yield). ^1^H-NMR gave broad peaks and not all quaternary centres were observed in the ^13^C-NMR. ^1^H-NMR (500 MHz, *d*_6_-DMSO): δ/ppm = 12.99 (s, 1H, NH), 11.15 (s, 1H, NH), 7.68 (d, br, *J =* 18 Hz, 2H), 7.51 (d, *J =* 9 Hz, 1H), 7.27 (dd, *J =* 6.5 Hz, 20.3 Hz, 2H), 7.15 (s, 1H), 2.50 (m, 3H), 2.49 (m, 3H). To separate broad peaks: ^1^H-NMR (400 MHz, *d*_6_-DMSO + 3 drops TFA): δ/ppm = 8.03 (1H from major regioisomer, d, *J =* 8.5 Hz, H3), 7.96 (1H from minor regioisomer, s, H1), 7.71 (1H from minor regioisomer, dd, *J =* 8.4 Hz, 1.9 Hz, H2), 7.48 (1H from major regioisomer, dd, *J =* 8.9 Hz, 1.3 Hz, H2), 7.41 (1H from minor regioisomer, d, *J =* 8.4 Hz, H3), 7.27 (1H from major regioisomer, s, H1) 2.62 (3H from major regioisomer, s, CH_3_), 2.56 (3H from minor regioisomer, s, CH_3_); ^13^C-NMR (125 MHz, *d*_6_-DMSO): δ/ppm = 163.7 (C), 154.5 (C), 153.2 (C), 143.7 (C), 134.1 (CH), 132.7 (C), 131.4 (C), 130.5 (C), 129.8 (CH), 129.5 (CH), 129.0 (C), 125.9 (CH), 115.5 (CH), 115.3 (CH), 21.6 (CH_3_), 20.4 (CH_3_). IR (neat) ν/cm^−1^: 3395.5 (w), 3134.2 (w, br), 1724.7 (s), 1693.2 (s), 1656.2 (m), 1626.1 (m), 1583.3 (m), 1533.1 (w), 1509.2 (m), 1465.3 (w), 1371.3 (s), 1290.6 (w), 1278.8 (w), 1248.7 (w), 1199.4 (w), 1185.1 (w), 1146.0 (w), 1116.6 (w), 1025.9 (w), 961.8 (w), 914.6 (w), 883.2 (w), 860.5 (w), 825.8 (s), 810.3 (s), 758.4 (w), 719.8 (w), 697.7 (w), 681.6 (w). LC-MS: R_t _3.49 min; HRMS (ESI): *m/z* calculated for C_11_H_9_N_4_O_3_ [M+H^+^]: 227.0574; found 227.0571.

*7-Methylbenzo[g]pteridine-2,4(3H,10H)-dione and 8-methyl-benzo[g]pteridine-2,4(3H,10H)-dione* (**7**). Prepared from 5-methyl-2-nitroaniline (0.152 g, 1 mmol) to give a yellow solid, with two regioisomers found in a 4:3 ratio (0.140 g, 61% yield). This was indicated by ^1^H-NMR to be identical to tne above compounds **7**.

*7-Methoxybenzo[g]pteridine-2,4(3H,10H)-dione and 8-methoxybenzo[g]pteridine-2,4(3H,10H)-dione* (**8**). Prepared from 4-methoxy-2-nitroaniline (0.206 g, 1 mmol) to give an orange solid, with two regioisomers found in a 3:2 ratio (0.1043 g, 43% yield). ^1^H-NMR (500 MHz, *d*_6_-DMSO): δ/ppm = 11.86 (1H from minor regioisomer, d, *J =* 1.7 Hz, NH), 11.82 (1H from major regioisomer, d, *J =* 1.7 Hz, NH), 11.69 (1H from major regioisomer, s, NH), 11.66 (1H from minor regioisomer, s, NH), 8.02 (1H from minor regioisomer, d, *J =* 9.2 Hz, H3), 7.83 (1H from major isomer, d, *J =* 9.2 Hz, H3), 7.59 (1H from major regioisomer, dd, *J =* 9.2 Hz, 2.7 Hz, H2), 7.54 (1H from major regioisomer, d, *J =* 2.8 Hz, H1), 7.40 (1H from minor regioisomer, dd, *J =* 9.3 Hz, 2.3 Hz, H2), 7.22 (1H from minor regioisomer, d, *J =* 2.7 Hz, H1), 3.97 (3H from minor regioisomer, s, CH_3_), 3.80 (3H from major regioisomer, s, CH_3_); ^13^C-NMR (125 MHz, *d*_6_-DMSO): δ/ppm = 163.25 (C), 160.7 (C), 160.6 (C), 159.1 (C), 150.3 (C), 150.1 (C), 147.2 (C), 145.5 (C), 145.1 (C), 145.1 (C), 140.8 (C), 138.8 (C), 135.6 (C), 131.5 (CH), 130.9 (C), 128.4 (C), 128.0 (CH), 126.9 (CH), 107.5 (CH), 104.9 (CH), 56.3 (CH_3_), 56.0 (CH_3_). IR (neat) ν/cm^−1^: 3258.9 (w), 3071.0 (w, br), 2851.2 (w), 1726.3 (m), 1694.1 (s), 1621.3 (w), 1556.8 (m), 1509.3 (w), 1458.9 (w), 1439.0 (m), 1399.2 (w), 1354.6 (s), 1340.8 (m), 1330.1(m), 1314.0 (w), 1233.5 (m), 1212.9 (s), 1158.8 (w), 1142.7 (w), 1120.4 (w), 1008.4 (m), 960.0 (w), 877.6 (w), 850.7 (m), 794.2 (m), 763.1 (m), 749.2 (m), 704.8 (w), 659.4 (w). LC-MS: R_t _3.42 min; HRMS (ESI): *m/z* calculated for C_11_H_7_N_4_O_3_ [M+H^+^]: 243.0524; found 243.0527.

*7-Fluorobenzo[g]pteridine-2,4(3H,10H)-dione and 8-Fluorobenzo[g]pteridine-2,4(3H,10H)-dione* (**9**). Prepared from 4-fluoro-2-nitroaniline (0.106 g, 1 mmol) to give a green solid with two regioisomers found in a 5:3 ratio (0.1678 g, 73% yield). ^1^H-NMR (500 MHz, *d*_6_-DMSO): δ/ppm = 12.04 (1H from minor regioisomer, d, *J =* 1.8 Hz, NH), 11.98 (1H from major regioisomer, d, *J =* 1.8 Hz, NH), 11.81 (1H from each regioisomer, br s, 2 × NH), 8.27 (1H from minor regioisomer, dd, *J =* 9.2 Hz, 6.1 Hz, H3), 8.02–7.99 (2H from major regioisomer, m, H1 and H2), 7.91–7.87 (1H from major regioisomer, m, H3), 7.74–7.69 (2H from minor regioisomer, m, H1 and H2); ^13^C-NMR (125 MHz, *d*_6_-DMSO): δ/ppm = 164.4 (C from minor regioisomer, d, *J =* 250.0 Hz, C-F), 164.4 (C from major regioisomer, d, *J =* 37.5 Hz, C-F), 160.4 (C), 160.3 (C), 150.1 (C, major regioisomer), 150.0 (C, minor regioisomer), 147.5 (C), 146.7 (C, d, *J =* 2.1 Hz), 143.9 (C from minor regioisomer, d, *J =* 14.4 Hz), 140.0 (C from major regioisomer), 139.5 (C from major regioisomer, d, *J =* 13.1 Hz), 136.6 (C), 133.0 (CH from minor regioisomer, d, *J =* 11.2 Hz, C3), 132.5 (C from major regioisomer), 131.3 (C from minor regioisomer, d, *J =* 3.0 Hz), 129.3 (CH from major regioisomer, d, *J =* 9.9 Hz, C3), 123.5 (CH from major regioisomer, d, *J =* 18.5 Hz, C2), 118.8 (CH from minor regioisomer, d, *J =* 26.2 Hz, C2), 113.3 (CH from major regioisomer, d, *J =* 21.5 Hz, C1), 110.7 (CH from minor regioisomer, d, *J =* 22.0 Hz, C1). IR (neat) ν/cm^−1^: 3182.7 (w), 3086.6 (w, br), 2845.9 (w), 1733.7 (m), 1691.9 (s), 1626.7 (m), 1583.9 (m), 1571.6 (m), 1510.4 (m), 1482.0 (w), 1455.3 (m), 1398.4 (w), 1354.5 (m), 1334.9 (s), 1298.6 (w), 1278.3 (s), 1243.1 (w), 1213.1 (s), 1157.4 (w), 1139.3 (w), 1108.9 (w), 1035.9 (w), 975.4 (w), 856.9 (s), 835.3 (s), 808.0 (m), 767.4 (m), 753.1 (s), 703.3 (w), 684.0 (w), 663.0 (m). LC-MS: R_t _2.85 min (mass peak of 233), another weaker peak seen at 3.45 min; HRMS (ESI): *m/z* calculated for C_10_H_4_N_4_O_2_F, [M+H^+^]: 231.0324; found 231.0323.

*7-(Trifluoromethyl)benzo[g]pteridine-2,4(3H,10H)-dione or 8-(trifluoromethyl)benzo[g]pteridine-2,4(3H,10H)-dione* (**10**). Prepared from 2-nitro-5-(trifluoromethyl)-aniline (0.206 g, 1 mmol) to give a pale yellow solid, one regioisomer was cleanly isolated (0.059 g, 21% yield). ^1^H-NMR (500 MHz, *d*_6_-DMSO): δ/ppm = 12.11 (br s, 1H, NH), 11.87 (br s, 1H, NH), 8.35 (d, *J =* 8.7 Hz, H3), 8.21 (d, *J =* 0.6 Hz, H1), 7.98 (dd, *J =* 8.7 Hz, 2.0 Hz, H2); ^13^C-NMR (125 MHz, *d*_6_-DMSO): δ/ppm = 160.1 (C), 150.1 (C), 148.0 (C), 141.7 (C), 140.2 (C), 134.4 (C) 132.3 (C-CF_3_) 132.0 (CH), 124.7 (CH), 124.0 (CF_3_), 123.4 (CH). IR (neat) ν/cm^−1^: 3456.0 (w), 3054.8 (w, br), 2898.1 (w), 2825.2 (w), 1733.4 (w), 1689.9 (s), 1631.4 (w), 1584.7 (w), 1507.7 (w), 1489.4 (w), 1454.8 (w), 1394.7 (m), 1367.9 (w), 1336.9 (w), 1318.3 (m), 1290.0 (s), 1266.5 (m), 1257.6 (m), 1244.0 (m), 1205.2 (m), 1166.9 (s), 1158.8 (s), 1136.1 (s), 1111.1 (m), 1086.1 (w), 1031.0 (w), 997.5 (m), 941.0 (w), 908.4 (w) 900.3 (w), 859.9 (s), 823.3 (w) 804.2 (m), 774.2 (w), 755.8 (w), 710.3 (s), 678.7 (w), 655.8 (w). LC-MS: R_t _3.85 min; HRMS (ESI): *m/z* calculated for C_11_H_4_F_3_N_4_O_2_ [M+H^+^]: 281.0292; found 281.0287.

*8-Chlorobenzo[g]pteridine-2,4(3H,10H)-dione and 7-chloro-benzo[g]pteridine-2,4(3H,10H)-dione* (**11**). Prepared from 4-chloro-2-nitroaniline (0.172 g, 1 mmol) to give a pale yellow solid, with two regioisomers found in a 5:1 ratio (0.121 g, 49% yield). ^1^H-NMR gave broad peaks. ^1^H-NMR (500 MHz, *d*_6_-DMSO): δ/ppm = 13.09 (1H from major regioisomer, br s, NH), 12.61 (1H from minor regioisomer, br s, NH), 10.98 (1H from major regioisomer, br s, NH), 8.25 (1H from minor regioisomer, s), 8.17 (1H from minor regioisomer, d, *J =* 9.0 Hz), 7.97–7.73 (4H, three from major regioisomer, one from minor regioisomer, br m). To separate broad peaks: ^1^H-NMR (400 MHz, *d*_6_-DMSO + 3 drops TFA): δ/ppm = 8.23 (1H from minor regioisomer, d, *J =* 1.5Hz, H1), 8.15 (1H from minor regioisomer, d, *J =* 9.0 Hz, H3), 7.93 (1H from major regioisomer, dd, *J =* 9.0 Hz, 2.1 Hz, H2), 7.74 (1H from minor regioisomer, dd, *J =* 9.0 Hz, 2.3 Hz, H2), 7.81 (1H from major regioisomer, m, H3), 6.70 (1H from major regioisomer, m, H1); ^13^C-NMR (125 MHz, *d*_6_-DMSO): δ/ppm = 160.3 (C), 153.1 (C), 150.1 (C), 147.6 (C), 147.2 (C), 143.1 (C), 141.4 (C), 141.4 (C), 139.4 (C), 137.8 (C), 133.7 (CH), 132.8 (C), 132.3 (C), 132.0 (CH), 131.7 (C), 131.4 (C), 128.6 (CH), 125.7 (CH), 117.5 (CH), 115.1 (CH). IR (neat) ν/cm^−1^: 3149.8 (w), 3085.3 (w, br), 2852.0 (w), 1728.1 (m), 1693.9 (s), 1661.4 (m), 1610.7 (m), 1578.6 (m), 1534.2 (w), 1490.7 (m), 1457.6 (w), 1365.9 (m), 1354.6 (s), 1335.6 (m), 1292.9 (m), 1274.7 (m), 1237.1 (w), 1190.2 (w), 1173.0 (w), 1145.2 (w), 1160.2 (w), 1082.7 (w), 1029.3 (w), 938.3 (w), 912.9 (w), 876.5 (w), 826.9 (m), 807.5 (m), 751.1 (w), 738.5 (w), 682.0 (w), 666.8 (w). LC-MS: R_t _3.60 min; HRMS (ESI): *m/z* calculated for C_10_H_4_N_4_O_2_Cl [M+H^+^]: 247.0028; found 247.0039.

*6,8-Dibromobenzo[g]pteridine-2,4(3H,10H)-dione or 7,9-dibromobenzo[g]pteridine-2,4(3H,10H)-dione* (**12**). Prepared from 2,4-dibromo-6-nitroaniline (0.294 g, 1 mmol) to give a pale green solid, one regioisomer was cleanly isolated (0.096 g, 26% yield). ^1^H-NMR (500 Hz, *d*_6_-DMSO): δ/ppm = 12.21 (s, 1H, NH), 11.85 (s, 1H, NH), 8.45 (d, *J =* 2.1 Hz, 1H), 8.43 (d, *J =* 2.1 Hz, 1H); ^13^C-NMR (125 MHz, *d*_6_-DMSO): δ/ppm = 159.9 (C), 150.0 (C), 147.8 (C), 139.9 (C), 139.4 (C), 138.2 (CH), 133.6 (C), 131.9 (CH), 122.0 (C), 120.2 (C). IR (neat) ν/cm^−1^: 3491.0 (w), 3183.1 (w) 3072.6 (w), 2849.1 (w), 1704.0 (s), 1603.2 (m), 1575.7 (w), 1558.3 (w), 1497.1 (w), 1468.2 (m), 1445.5 (m), 1386.8 (m), 1347.8 (m), 1319.7 (w), 1286.5 (s), 1210.7 (w), 1181.7 (w), 1136.6 (w), 1076.6 (w), 1028.2 (m), 1010.7 (m), 949.6 (m), 869.9 (m), 833.0 (m), 814.7 (m), 750.9 (w), 732.6 (w), 710.1 (w), 670.6 (w). LC-MS: R_t _4.02 min; HRMS (ESI): *m/z* calculated for C_10_H_3_N_4_O_2_Br_2_ [M+H^+^]: 368.8642; found 368.8628.

### 3.4. General Procedure for the Formation of the Dehydroquinoxalines Analogues **14**–**22**

The diamines generated by the hydrogenation process in flow were mixed with alloxane monohydrate (0.160 g, 1 mmol) and left to stir at room temperature overnight. The product was then filtered and the solid product was dried *in vacuo*.

*N-Carbamoyl-6,7-dimethyl-3-oxo-3,4-dihydroquinoxaline-2-carboxamide* (**14**). Prepared from 4,5-dimethyl-2-nitroaniline (0.166 g, 1 mmol) to give a dull yellow solid (0.212 g, 84% yield). ^1^H-NMR (500 MHz, *d*_6_-DMSO): δ/ppm = 12.97 (br s, 1H, NH), 11.21 (br s, 1H, NH), 7.74 (br s, 1H, NH from NH_2_), 7.69–7.65 (br m, 2H), 7.54 (br s, 1H, NH from NH_2_). To separate broad peaks: ^1^H-NMR (400 MHz, *d*_6_-DMSO + 3 drops TFA): δ/ppm = 6.55 (s, 1H), 6.45 (s, 1H), 5.72 (s, 2H from NH_2_); ^13^C-NMR (125 MHz, *d*_6_-DMSO): δ/ppm = 159.6 (C), 155.2 (C), 153.2 (C), 142.0 (C), 141.9 (C), 131.2 (C), 128.8 (C), 123.4 (C), 115.8 (CH), 115.6 (CH), 20.2 (CH_3_), 19.0 (CH_3_). IR (neat) ν/cm^−1^: 3377.1 (m), 3148.1 (w, br), 2823.3 (w, br), 1690.1 (s), 1645.9 (m), 1570.3 (s), 1487.5 (s), 1446.3 (s), 1390.6 (s), 1390.6 (s), 1367.5 (s), 1331.5 (m), 1286.8 (w), 1253.5 (m), 1196.5 (m), 1167.9 (s), 1111.9 (m), 1032.3 (w), 1011.4 (w), 967.3 (w), 919.9 (w), 860.8 (w), 820.5 (w), 801.8 (s), 752.8 (w), 741.6 (w), 683.7 (m). LC-MS: R_t_ 3.69 min; HRMS (ESI): *m/z* calculated for C_12_H_11_N_4_O_3_ [M+H^+^]: 259.0837; found 259.0841.

*N-Carbamoyl-3-oxo-3,4-dihydroquinoxaline-2-carboxamide* (**15**). Prepared from 2-nitroaniline (0.138 g, 1 mmol) to give a light green solid (0.228 g, 98% yield). ^1^H-NMR gave broad peaks. ^1^H-NMR (500 MHz, *d*_6_-DMSO): δ/ppm = 13.03 (s, 1H, NH), 11.09 (s, 1H, NH), 7.92–7.37 (m br, 6H, NH_2_ + 4CH). To separate broad peaks: ^1^H-NMR (400 MHz, *d*_6_-DMSO + 3 drops TFA): δ/ppm = 8.97 (br s, 1H, NH_2_), 7.37 (br s, 1H, NH_2_), 6.83 (m, 2H), 6.68 (m, 2H); ^13^C-NMR (125 MHz, *d*_6_-DMSO): δ/ppm = 163.8 (C), 154.1 (C), 153.2 (C), 149.3 (C), 132.6 (CH), 131.3 (C), 129.7 (CH), 124.3 (CH), 124.0 (C), 115.8 (CH). IR (neat) ν/cm^−1^: 3366.5 (w), 3152.2 (w, br), 2969.0, (w), 2813.8 (w, br), 1718.3 (m), 1685.2 (s), 1643.2 (m), 1609.6 (w), 1571.0 (m), 1495.8 (m), 1433.8 (m), 1391.0 (s), 1360.1 (m), 1336.6 (w), 1293.6 (w), 1270.1 (w), 1249.6 (w), 1232.8 (w), 1183.2 (w), 1146.8 (m), 1100.7 (m), 1049.0 (w), 969.8 (w), 929.1 (w), 912.1 (m), 796.1 (m), 764.1 (s), 723.9 (w), 680.4 (w). LC-MS: R_t _2.63 min; HRMS (ESI): *m/z* calculated for C_10_H_7_N_4_O_3_ [M+H^+^]: 213.0524; found 231.0527.

*N-Carbamoyl-7-methyl-3-oxo-3,4-dihydroquinoxaline-2-carboxamide and N-carbamoyl-8-methyl-3-oxo-3,4-dihydroquinoxaline-2-carboxamide* (**16**). Prepared from 4-methyl-2-nitroaniline (0.152 g, 1 mmol) to give a bright yellow solid, with two regioisomers in a 3:2 ratio (0.187 g, 76% yield). ^1^H-NMR (500 MHz, *d*_6_-DMSO): δ/ppm = 11.86 (1H from minor regioisomer, d, *J =* 1.7 Hz, NH), 11.84 (1H from major regioisomer, d, *J =* 1.7 Hz, NH) 11.68 (1H from major regioisomer, s, NH), 11.69 (1H from minor regioisomer, s, NH) , 8.00 (1H from minor regioisomer, d, *J =* 8.6 Hz, H1) 7.89 (1H from major regioisomer, s, H3), 7.78 (1H from major regioisomer, d, *J =* 8.6 Hz, H1), 7.72 (1H from major isomer, dd, *J =* 8.6 and 1.9 Hz, H2), 7.66 (1H from minor regioisomer, s, H3), 7.57 (1H from minor regioisomer, dd, *J =* 8.6 and 1.9 Hz, H2), 2.54 (3H from minor regioisomer, s, CH_3_), 2.48 (3H from major regioisomer, m, CH_3_). ^13^C-NMR (125 MHz, *d*_6_-DMSO): δ/ppm = 160.6 (C), 160.6 (C), 150.2 (C), 150.1 (C), 147.0 (C), 146.4 (C), 144.3 (C), 142.8 (C), 141.1 (C), 139.4 (C), 138.6 (C), 137.9 (C), 135.7 (CH), 131.3 (C), 130.8 (CH), 130.6 (C), 129.7 (CH), 128.7 (CH), 126.6 (CH), 125.7 (CH), 21.7 (CH_3_), 21.1 (CH_3_). IR (neat) ν/cm^−1^: 3171.9 (w), 3075.1 (w), 2990.5 (w),2852.3 (w), 1726.5 (m), 1701.1 (s), 1622.7 (w), 1581.9 (m), 1564.7 (w), 1511.5 (w), 1476.0 (w), 1446.6 (w), 1392.9 (w), 1354.4 (m), 1337.5 (m), 1307.9 (w), 1281.0 (s), 1249.4 (w), 1207.9 (w), 1151.2 (w), 1121.8 (w), 1027.5 (w), 982.2 (w), 948.9 (w), 907.4 (w), 874.6 (w), 830.1 9s), 810.0 (m), 760.8 (w), 704.4 (w), 682.0 (w), 661.8 (w). LC-MS: R_t _3.43 min; HRMS (ESI): *m/z* calculated for C_11_H_9_N_4_O_3_ [M+H^+^]: 245.0680; found 245.0681.

*N-Carbamoyl-7-methyl-3-oxo-3,4-dihydroquinoxaline-2-carboxamide and N-carbamoyl-8-methyl-3-oxo-3,4-dihydro-quinoxaline-2-carboxamide* (**17**). Prepared from 5-methyl-2-nitroaniline (0.152 g, 1 mmol) to give a bright yellow solid, with two regioisomers found in a 3:2 ratio (0.215 g, 87% yield). This was gave an identical ^1^H-NMR to **16**.

*N-Carbamoyl-7-methoxy-3-oxo-3,4-dihydroquinoxaline-2-carboxamide and N-carbamoyl-8-methoxy-3-oxo-3,4-dihydroquinoxaline-2-carboxamide* (**18**). Prepared from 4-methoxy-2-nitroaniline (0.206 g, 1 mmol) to give an orange solid, with two regioisomers found in a 5:2 ratio (0.231 g, 88% yield). ^1^H-NMR gave broad peaks and not all quaternary centres were observed in the ^13^C-NMR. ^1^H-NMR (500 MHz, *d*_6_-DMSO): δ/ppm = 13.03 (s, 1H, NH), 11.23 (s, 1H, NH), 7.83-7.30 (br m, 6H), 7.03 (d, *J =* 8.7 Hz, 2H, NH_2_), 6.81 (d, *J =* 2.9 Hz, 2H, NH_2_), 3.88 (s, 3H, CH_3_), 3.84 (s, 3H, CH_3_). To separate broad peaks: ^1^H-NMR (400 MHz, *d*_6_-DMSO + 3 drops TFA): δ/ppm = 7.79 (1H from minor regioisomer, d, *J =* 9.0 Hz, CH, 2 or 3), 7.58 (1H from major regioisomer, s, H1), 7.00 (1H from minor regioisomer, d, *J =* 9.5 Hz, H2 or H3), 6.81 (1H from minor regioisomer, d, *J =* 2.5 Hz, H1) 6.69 (1H from major regioisomer, d, *J =* 9.3 Hz, H2 or H3), 6.60 (1H from major regioisomer, d, *J =* 9.3 Hz, H2 or H3), 6.44 (2H, one from each regioisomer, d, *J =* 6.9 Hz, 2NH from the NH_2_), 6.24 (2H, one from each regioisomer, d, *J =* 6.6 Hz, 2NH from the NH_2_); ^13^C-NMR (125 MHz, *d*_6_-DMSO): δ/ppm = 162.9 (C), 153.2 (C), 153.2 (C), 134.89 (C), 131.6 (CH), 126.9 (C), 116.7 (CH), 114.1 (CH), 110.5 (CH), 103.7 (CH), 97.6 (CH), 56.0 (CH_3_), 55.8 (CH_3_). IR (neat) ν/cm^−1^: 3394.4 (w), 3315.5 (w), 3149.3 (w,br), 2976.0 (w), 1731.0 (s), 1697.5 (s), 1676.5 (w), 1648.2 (s), 1618.5 (s), 1583.6 (s), 1502.4 (s) 1487 (s), 1471.4 (m), 1455.1 (m), 1441.4 (w), 1381.8 (s), 1370.6 (s)., 1280.7 (w), 1249.7 (m), 1222.3 (s), 1184.2 (s), 1150.6 (m), 1110.4 (m), 1031.7 (m), 1012.8 (m), 969.4 (w), 914.1 (w), 839.5 (s), 822.0 (s), 801.6 (s), 751.8 (w), 682.0 (w). LC-MS: R_t _3.44 min; HRMS (ESI): *m/z* calculated for C_11_H_9_N_4_O_4_ [M+H^+^]: 261.0629; found 261.0634.

*N-Carbamoyl-7-fluoro-3-oxo-3,4-dihydroquinoxaline-2-carboxamide and N-carbamoyl-8-fluoro-3-oxo-3,4-dihydro-quinoxaline-2-carboxamide* (**19**). Prepared from 4-fluoro-2-nitroaniline (0.106 g, 1 mmol) to give a pale green solid, with two regioisomers found in a 5:1 ratio (0.165 g, 66% yield). ^1^H-NMR gave broad peaks and not all quaternary centres were observed in the ^13^C-NMR. ^1^H-NMR (500 MHz, *d*_6_-DMSO): δ/ppm = 13.07 (1H from major regioisomer, br s, NH), 12.55 (1H from minor regioisomer, br s, NH), 11.03 (1H from major regiosiomer, br s, NH), 10.34 (1H from minor regioisomer, br s, NH), 7.94 (1H from major regioisomer, br s, NH from NH_2_) 7.76–7.57 (5H from minor regioisomer and 1H from major regioisomer, br m, 2NH from NH_2_, 4CH), 7.38 (1H from major regioisomer, br s, NH from NH_2_), 7.25 (1H from major regioisomer, s), 7.07 (1H from major regioisomer, d, *J =* 4.1 Hz); ^13^C-NMR (125 MHz, *d*_6_-DMSO): δ/ppm = 163.7 (C), 159.1 (C), 157.2 (C), 154.0 (C-F), 134.4 (C), 132.4 (CH), 131.5 (C), 129.6 (CH), 128.4 (C), 121.0 (CH), 117.4 (C), 114.5 (CH), 112.6 (CH), 101.6 (CH). IR (neat) ν/cm^−1^: 3380.2 (w), 3152.2 (w, br), 2732.4 (w,br) 1721.4 (s), 1698.5 (s), 1649.5 (s), 1596.5 (m), 1578.9 (m), 1504.1 (s), 1485.6 (s), 1403.6 (s), 1364.1 (s), 1284.1 (w), 1246.3 (m), 1201.8 (m), 1179.4 (m), 1144.7 (w), 1120.1 (w), 1099.3 (m), 978.9 (w), 939.0 (w), 912.9 (w), 847.0 (m), 823.2 (s), 805.3 (s), 752.8 (w), 698.9 (w), 680.7 (m). LC-MS: R_t _3.23 min; HRMS (ESI): *m/z* calculated for C_10_H_6_N_4_O_3_F [M+H^+^]: 249.0429; found 249.0421.

*N-Carbamoyl-3-oxo-7(trifluoromethyl)-3,4-dihydro-quinoxaline-2-carboxamide and N-carbamoyl-3-oxo-8(trifluoromethyl)-3,4-dihydroquinoxaline-2-carboxamide* (**20**). Prepared from 2-nitro-5-(trifluromethyl)-aniline (0.206 g, 1 mmol) to give a pale yellow solid, with two regioisomers in a 5:2 ratio (0.178 g, 59% yield). ^1^H-NMR gave broad peaks and not all quaternary centres were observed in the ^13^C-NMR. ^1^H-NMR (500 MHz, *d*_6_-DMSO): δ/ppm = 13.21 (1H from major regioisomer, s, NH), 12.78 (1H from minor regioisomer, br d, *J =* 40.35 Hz, NH), 10.95 (1H from major regioisomer, s, NH), 10.39 (1H from minor regioisomer, br, s, NH), 8.23–7.51 (6H, three from each regioisomer, m), 6.96 (2H, one from each regioisomer, br s, 2NH from the NH_2_’s), 6.23 (2H, one from both compounds, br s, 2NH from the NH_2_’s). To separate broad peaks: ^1^H-NMR (400 MHz, *d*_6_-DMSO + 3 drops TFA): δ/ppm = 7.97 (1H from minor regioisomer, s, NH from NH_2_), 7.92 (1H from minor regioisomer, s, NH from NH_2_), 7.76 (1H from minor regioisomer, s, H1), 7.62 (1H from minor regioisomer, s, H2 or H3), 7.51 (1H from minor regioisomer, d, *J =* 8.4 Hz, H2 or H3), 7.17 (1H from major regioisomer, d, *J =* 8.3, H2 or H3), 7.07 (1H from major regioisomer, s, H1), 7.01 (1H from major regioisomer, d, *J =* 8.3 Hz, NH from NH_2_), 6.96 (1H from major regioisomer, d, *J =* 8.3 Hz, NH from NH_2_), 6.81 (1H from major regioisomer, *J =* 8.3 Hz, H2 or H3); ^13^C-NMR (125 MHz, *d*_6_-DMSO): δ/ppm = 166.5 (CF_3_), 163.8 (C), 153.0 (C-CF_3_), 135.4 (C), 133.0 (C), 130.9 (CH), 130.4 (C), 128.4 (CH), 126.9 (CH), 125.1 (C), 124.7 (C, minor regioisomer), 122.8 (C, minor regioisomer), 122.5 (C, minor regioisomer), 120.1 (CH), 117.2 (CH), 113.0 (CH). IR (neat) ν/cm^−1^: 3520.9 (w), 3411.4 (w), 3243.7 (w), 3159.1 (w), 1736.8 (m), 1692.7 (s), 1674.6 (s), 1662.0 (s), 1625.9 (m), 1568.0 (w), 1506.1 (w), 1466.7 (w). 1412.1 (w), 1376.5 (m), 1320.3 (m), 1285.7 (w), 1260.6 (w), 1242.3 (w), 1189.4 (w), 1166.3 (w), 1148.9 (s), 1135.7 (s), 1112.7 (m), 1091.4 (m), 1064.7 (m), 957.1 (w),901.4 (m), 847.0 (m), 832.5 (m), 820.7 (w), 779.8 (w), 738.9 (w), 687.3 (m), 655.3 (m). LC-MS: R_t _3.80 min; HRMS (ESI): *m/z* calculated for C_11_H_4_F_3_N_4_O_2_ [M+H^+^]: 281.0292; found 281.0287.

*N-Carbamoyl-6-chloro-3-oxo-3,4-dihydroquinoxaline-2-carboxamid and N-carbamoyl-7-chloro-3-oxo-3,4-dihydro-quinoxaline-2-carboxamide* (**21**). Prepared from 4-chloro-2-nitroaniline (0.172 g, 1 mmol) to give a pale yellow solid, with two regioisomers in an unknown ratio due to overlapping ^1^H signals (0.157 g, 59% yield). ^1^H-NMR (500 MHz, *d*_6_-DMSO): δ/ppm = 12.02 (s, 2H, 2NH), 11.79 (s, 2H, 2NH), 8.17 (d, *J =* 9.0 Hz, 2xH3), 7.97 (d, *J =* 2.3 Hz, 2 × H1), 7.93 (4H, m, 2H, 2NH*_2_*), 7.78 (dd, *J =* 2.3 Hz, 9.0 Hz, 2 × H2); ^13^C-NMR (125 MHz, *d*_6_-DMSO): δ/ppm = 160.3 (br, 2C), 150.1 (br, C), 147.6 (C), 147.2 (C), 143.1 (C), 141.4 (C), 139.4 (C), 137.9 (C), 137.8 (C), 133.7 (CH), 132.8 (C), 132.5 (C), 132.3 (C), 132.0 (CH), 129.1 (CH), 128.8 (CH), 128.6 (CH), 125.7 (CH). IR (neat) ν/cm^−1^: 3457.6 (w, br), 3184.9 (w), 3068.7 (w, br), 2850.5 (w), 1727.7 (m), 1697.9 (s), 1614.5 (m), 1578.8 (m), 1562.0 (w), 1489.7 9(w), 1449.0 (w), 1388.9 (m), 1354.7 (m), 1335.8 (m), 1292.5 (m), 1275.0 (m), 1245.9 (w), 1196.3 (w), 1145.6 (w), 1112.4 (w), 1071.0 (w), 1029.8 (m), 939.2 (w), 876.7 (w), 847.5 (s), 806.6 (m), 751.5 (m), 738.1 (m), 692.8 (w), 666.2 (w). LC-MS: R_t _3.60 min; HRMS (ESI): *m/z* calculated for C_10_H_4_N_4_O_2_Cl [M+H^+^]: 266.0207; found 266.0211.

*6,8-Dibromo-N-carbamoyl-3-oxo-3,4-dihydroquinoxaline-2-carboxamide or 7,9-dibromo-N-carbamoyl-3-oxo-3,4-dihydroquinoxaline-2-carboxamide* (**22**). Prepared from 2,4-dibromo-6-nitroaniline (0.294 g, 1 mmol) to give a mixture of a green solid and a liquid component in approximately a 1:2 ratio. Upon purification one regioisomer was cleanly isolated (estimated yield 8%). HRMS (ESI): *m/z* calculated for C_10_H_5_N_4_O_3_Br_2_ [M+H^+^]: 386.8734; found 386.8716. ^1^H-NMR (400 MHz, *d*_6_-DMSO): δ/ppm = 11.20 (s, 1H, NH), 11.04 (s, 1H, NH), 8.16 (d, *J =* 2.6 Hz, 1H), 8.05 (d, *J =* 2.2 Hz, 1H).

### 3.5. General Procedure for the Formation of 1,5-Dibenzodiazepines **27a**–**k**

A mixture of the fluoro nitrobenzene derivative and the corresponding amino benzophenone/fluorenone (1:1) was dissolved in dry THF in a microwave vial. Lithium bis(trimethylsilyl)amide (LiHMDS, 1.0 M solution in THF) was added dropwise and the mixture heated to 100 °C for 30 to 90 min using a Biotage Initiator microwave instrument. The reaction was quenched by addition of water and the mixture extracted with EtOAc. The combined organic layers were dried over anhydrous Na_2_SO_4_ and the solvent evaporated under vacuum. Most of the intermediate products (**25**) were not further purified but directly hydrogenated in flow, although some of them were subjected to column chromatography and fully characterised to confirm the structure and complete data is shown below.

*(2-((5-Chloro-2-nitrophenyl)amino)phenyl)(phenyl)-methanone.* ^1^H-NMR (400 MHz, CDCl_3_): δ/ppm = 10.98 (s, 1H, NH), 8.09 (d, *J =* 8.8 Hz, 1H), 7.81 (d, *J =* 1.1 Hz, 1H), 7.79 (d, *J =* 1.5 Hz, 1H), 7.62–7.56 (m, 4H), 7.47–7.44 (m, 3H), 7.20 (dd, *J =* 7.6 Hz, 1.5 Hz, 1H), 6.80 (dd, *J =* 9.0 Hz, 2.0 Hz, 1H); ^13^C-NMR (100 MHz, CDCl_3_): δ 196.7 (C=O), 141.7 (C), 141.3 (C), 139.7 (C), 138.1 (C), 134.2 (C), 133.3 (CH), 133.3 (2 × CH), 130.5 (2 × CH), 129.8 (C), 128.8 (2 × CH), 128.5 (CH), 123.8 (CH), 122.5 (CH), 119.7 (CH), 117.1 (CH). IR (neat) ν/cm^−1^: 3301.0 (w), 3062.6 (w), 1643 (w), 1608.2 (m), 1596.2 (m), 1562.6 (s), 1486.6 (s), 1449.3 (m), 1409.4 (w), 1312.5 (m), 1335.1 (w), 1297.1 (m), 1248.8 (s), 1211.5 (m), 1180.2 (w), 1164.3 (w), 1103.8 (w), 1070.6 (w), 921.6 (m), 841.4 (w), 749.9 (m), 700.8 (m). HRMS (*m/z*) calculated for C_19_H_14_O_3_N_2_Cl, (M-H), 353.0687; found 353.0678

*(5-Chloro-2-((5-chloro-2-nitrophenyl)amino)phenyl)-(phenyl)methanone.* ^1^H-NMR (400 MHz, CDCl_3_): δ/ppm = 10.82 (s, 1H, NH), 8.11 (d, *J =* 9.2 Hz, 1H), 7.82 (d, *J =* 1.5 Hz, 1H), 7.80 (d, *J =* 1.4 Hz, 1H), 7.65–7.58 (m, 2H), 7.56 (s, 1H), 7.54 (d, *J =* 2.0 Hz, 1H), 7.53–7.48 (m, 2H), 7.40 (d, *J =* 2.0 Hz, 1H), 6.80 (dd, *J =* 9.2 Hz, 2.2 Hz, 1H); ^13^C-NMR (100 MHz, CDCl_3_): δ 195.3 (C=O), 141.8 (C), 140.9 (C), 138.2 (C), 137.1 (C), 134.1 (C), 133.4 (CH), 132.8 (CH), 132.2 (CH), 130.9 (C), 130.1 (2 x CH), 128.7 (C), 128.6 (2 × CH), 128.2 (CH), 123.6 (CH), 119.7 (CH), 116.6 (CH). IR (neat) ν/cm^−1^: 2673.4 (w), 2319.2 (w), 1646.9 (w), 1607.9 (m), 1568.3 (m), 1488.2 (s), 1395.9 (w), 1336.5 (w), 1294.2 (w), 1259.7 (m), 1240.5 (m), 1163.3 (w), 1104.2 (w), 930.4 (w), 826.6 (w), 752.2 (w). HRMS (*m/z*) calculated for C_19_H_13_O_3_N_2_Cl_2_, (M-H), 387.0298; found 387.0289.

*(5-Chloro-2-((5-chloro-2-nitrophenyl)amino)phenyl)(3-chlorophenyl)methanone.* ^1^H-NMR (400 MHz, CDCl_3_): δ/ppm = 11.33 (s, 1H, NH), 8.14 (d, *J =* 9.1 Hz, 1H), 7.57–7.40 (m, 8H), 6.94 (dd, *J =* 9.1 Hz, 2.2 Hz, 1H); ^13^C-NMR (100 MHz, CDCl_3_): δ 195.7 (C=O), 140.2 (C), 140.1 (C), 138.3 (C), 135.8 (C), 134.6 (CH), 133.5 (CH), 132.4 (CH), 131.8 (C), 130.7 (CH), 130.0 (CH), 128.5 (CH), 128.5 (C), 128.2 (C), 127.4 (CH), 122.2 (CH), 121.1 (CH), 118.3 (CH). IR (neat) ν/cm^−1^: 2738.9 (w), 1648.0 (w), 1607.8 (m), 1570.8 (m), 1488.2 (s), 1397.9 (w), 1336.5 (w), 1293.2 (s), 1247.5 (m), 1072.3 (w), 951.9 (w), 929.7 (w), 828.3 (w), 748.9 (w), 699.8 (w). HRMS (*m/z*) calculated for C_19_H_11_O_3_N_2_Cl_3_Na, 442.9727; found 442.9724.

*5-Chloro-2-((5-chloro-2-nitrophenyl)amino)phenyl)(2-fluoro-phenyl)methanone.* ^1^H-NMR (400 MHz, CDCl_3_): δ/ppm = 11.09 (s, 1H, NH), 8.11 (d, *J =* 9.2 Hz, 1H), 7.64–7.48 (m, 5H), 7.44 (d, *J =* 2.2 Hz, 1H), 7.28 (td, *J =* 7.7 Hz, 1.1 Hz, 1H), 7.13 (*app* t, 1H), 6.88 (dd, *J =* 9.0 Hz, 2.0 Hz, 1H); ^13^C-NMR (100 MHz, CDCl_3_): δ 192.7 (C=O), 160.1 (d, *J =* 253.6 Hz, C-F), 141.7 (C), 140.4 (C), 138.6 (C), 134.8 (C), 134.3 (d, *J =* 8.4 Hz, CH), 133.7 (CH), 132.5 (d, *J =* 2.3 Hz, CH), 131.0 (d, *J =* 1.9 Hz, CH), 130.2 (C), 128.5 (CH), 128.1 (C), 126.4 (d, *J =* 13.6 Hz, C), 124.7 (d, *J =* 3.6 Hz, CH), 122.6 (CH), 120.2 (CH), 117.4 (CH), 116.5 (d, *J =* 21.6 Hz, CH). IR (neat) ν/cm^−1^: 2338.9 (w), 1648.9 (w), 1608.6 (m), 1570.1 (m), 1488.6 (s), 1453.1 (w), 1398.5 (w), 1336.3 (w), 1308.8 (w), 1259.7 (m), 1243.7 (m), 1213.9 (w), 1072.1 (w), 931.2 (w), 753.12 (m). HRMS (*m/z*) calculated for C_19_H_12_O_3_N_2_Cl_2_F (M+H), 405.0204; found 405.0197.

The nitro phenylamino derivatives **25** were hydrogenated in flow at 60 °C using a flow rate of 0.5 mL/min, following the general procedure described before. The generated diamines were collected over MgSO_4_ and stirred for a further 1–2 h at room temperature to enhance the cyclisation process. After filtration to remove the drying agent, catalytic formic acid was added to the remaining solution and the mixture was stirred at room temperature overnight. The solvent was then removed under vacuum and the final benzodiazepines were purified by column chromatography and fully characterised by NMR.

*11-Phenyl-5H-dibenzo[b,e][1,4]diazepine* (**27a**)*.* Prepared from 1-fluoronitrobenzene and 2-aminobenzophenone (175 mg, 65% yield). ^1^H-NMR (600 MHz, CDCl_3_): δ/ppm = 7.76 (d, *J =* 7.1 Hz, 2H), 7.48–7.42 (m, 3H), 7.36 (dd, *J =* 7.7 Hz, 1.0 Hz, 1H), 7.29 (td, *J =* 7.6 Hz, 1.1 Hz, 1H), 7.08 (td, *J =* 7.5 Hz, 1.0 Hz, 1H), 7.04 (m, 2H), 6.94 (t, *J =* 7.5 Hz, 1H), 6.77 (d, *J =* 7.9 Hz, 1H), 6.70 (dd, *J =* 7.6 Hz, 0.8 Hz, 1H), 5.03 (s, 1H); ^13^C-NMR (150 MHz, CDCl_3_): δ 169.6 (C), 154.5 (C), 142.7 (C), 141.4 (C), 140.9 (C), 132.2 (CH), 132.0 (CH), 130.0 (CH), 129.6 (2xCH), 128.7 (CH), 128.0 (2 × CH), 127.6 (C), 126.9 (CH), 124.2 (CH), 122.4 (CH), 119.8 (2 × CH); IR (neat) cm^−1^: 3354.0 (w), 3273.2 (w), 3051.8 (w), 2850.2 (w), 1601.9 (w, N=C), 1494.8 (w), 1460.2 (m), 1401.3 (w), 1317.0 (w), 1283.1 (w), 1234.6 (w), 1151.7 (w), 1108.2 (w), 958.5 (w), 944.3 (w), 853.1 (w), 802.7 (w), 756.4 (s), 694.7 (s), 657.7 (m); LC-MS: *t_r_* = 4.68, *m/z* = 271.32 (C_19_H_14_N_2_)^+^.

*7-Chloro-11-phenyl-5H-dibenzo[b,e][1,4]diazepine* (**27b**). Prepared from 4-chloro-2-fluoro-1-nitrobenzene and 2-aminobenzophenone (201 mg, 66% yield). ^1^H-NMR (600 MHz, CDCl_3_): δ/ppm = 7.70 (d, *J =* 7.1 Hz, 2H), 7.47–7.40 (m, 3H), 7.32 (t, *J =* 7.0 Hz, 1H), 7.22 (d, *J =* 8.3 Hz, 1H), 7.02 (d, *J =* 8.0 Hz, 2H), 6.97 (t, *J =* 7.4 Hz, 1H), 6.77 (d, *J =* 7.8 Hz, 1H), 6.72 (d, *J =* 1.8 Hz, 1H), 4.97 (s, 1H); ^13^C-NMR (150 MHz, CDCl_3_): δ 169.8 (C), 153.6 (C), 143.4 (C), 141.0 (C), 139.5 (C), 132.3 (C), 132.2 (CH), 132.1 (CH), 130.2 (CH), 129.6 (CH), 129.5 (2 × CH), 128.0 (2xCH), 127.5 (C), 124.2 (CH), 122.8 (CH), 119.9 (CH), 119.7 (CH); IR (neat) cm^−1^: 3359.8 (w, NH), 3053.7 (w), 1607.9 (s, N=C), 1573.1 (w), 1455.8 (s), 1433.3 (w), 1317.6 (w), 1285.6 (m), 1250.0 (w), 1117.5 (w), 1089.7 (w), 957.6 (w), 915.0 (m), 855.7 (m), 815.9 (s), 778.8 (s), 742.5 (s), 722.6 (m), 692.8 (s), 681.2 (s), 666.1 (m); LC-MS: *t_r_* = 5.13, *m/z* = 304.90 (C_19_H_13_ClN_2_)^+^. The structure was unambiguously confirmed by X-ray crystallography and deposited at the Cambridge Crystallographic Data Centre with the unique reference number CCDC 867823; Formula: C_19_H_13_ClN_2_, unit cell parameters: *a* = 16.4748(5) Å, *b* = 5.6296(2) Å, *c* = 16.0146(7) Å, *α* = 90°, *β* = 102.528(2)°, *γ* = 90°, space group: *P*2_1_/*c*.

*2-Chloro-11-phenyl-5H-dibenzo[b,e][1,4]diazepine* (**27c**). Prepared from 1-fluoronitrobenzene and 2-amino-4-chlorobenzophenone (110 mg, 72% yield). ^1^H-NMR (600 MHz, CDCl_3_): δ/ppm = 7.71 (d, *J =* 7.1 Hz, 2H), 7.49-7.42 (m, 3H), 7.32 (dd, *J =* 7.6 Hz, 1.4 Hz, 1H), 7.27–7.25 (m, 1H), 7.09–7.03 (m, 2H), 6.99 (d, *J =* 2.3 Hz, 1H), 6.74 (d, *J =* 8.5 Hz, 1H), 6.71 (dd, *J =* 7.5 Hz, 1.3 Hz, 1H), 5.02 (s, 1H); ^13^C-NMR (150 MHz, CDCl_3_): δ 153.0 (C), 142.2 (C), 140.5 (C), 140.4 (C), 131.8 (CH), 131.6 (CH), 130.2 (CH), 129.5 (2 × CH), 128.8 (CH), 128.2 2 × (CH), 127.9 (C), 127.8 (C), 127.7 (C), 127.2 (CH), 124.5 (CH), 121.1 (CH), 119.8 (CH); IR (neat) cm^−1^: 3269.5 (w, NH), 3053.0 (w), 1610.6 (w, N=C), 1569.3 (w), 1489.0 (w), 1461.7 (w), 1421.2 (w), 1318.9 (w), 1280.2 (w), 1234.7 (w), 1120.4 (w), 964.9 (w), 823.2 (m), 806.3 (m), 760.2 (s), 738.6 (s), 698.1 (s), 667.8 (w); LC-MS: *t_r_* = 5.09, *m/z* = 305.03 (C_19_H_13_ClN_2_)^+^.

*2,7-Dichloro-11-phenyl-5H-dibenzo[b,e][1,4]diazepine* (**27d**). Prepared from 4-chloro-2-fluoro-1-nitrobenzene and 2-amino-4-chlorobenzophenone (99 mg, 59% yield). ^1^H-NMR (600 MHz, CDCl_3_): δ/ppm = 7.69 (d, *J =* 7.2 Hz, 2H), 7.49-7.40 (m, 3H), 7.27 (m, 1H), 7.22 (d, *J =* 8.4 Hz, 1H), 7.04 (dd, *J =* 8.4 Hz, 2.2 Hz, 1H), 6.99 (d, *J =* 2.3 Hz, 1H), 6.73–6.71 (m, 2H), 4.99 (s, 1H); ^13^C-NMR (150 MHz, CDCl_3_): δ 168.2 (C), 152.0 (C), 142.9 (C), 140.3 (C), 139.2 (C), 132.4 (C), 131.9 (CH), 131.6 (CH), 130.5 (CH), 129.7 (CH), 129.6 (2 × CH), 129.4 (C), 128.9 (C), 128.3 (2 × CH), 124.5 (CH), 121.2 (CH), 119.8 (CH); IR (neat) cm^−1^: 3359.6 (w), 3259.7 (w), 3054.2 (w), 1608.7 (m, N=C), 1573.9 (w), 1454.7 (m), 1316.9 (m), 1287.3 (w), 1249.8(w), 1117.0 (w), 1087.6 (w), 957.9 (w), 915.2 (m), 858.1 (m), 816.5 (m), 778.8 (m), 742.8 (m), 691.9 (s); LC-MS: *t_r_* = 5.35, *m/z* = 339.22 (C_19_H_12_Cl_2_N_2_)^+^. The structure was unambiguously confirmed by X-ray crystallography and deposited at the Cambridge Crystallographic Data Centre with the unique reference number CCDC 867824; Formula: C_19_H_12_Cl_2_N_2_, unit cell parameters: *a* = 14.2135(5) Å, *b* = 9.2512(4) Å, *c* = 25.0981(10) Å, *α* = 90°, *β* = 103.764(2)°, *γ* = 90°, space group: C2/*c*.

*2,7-Dichloro-11-(2-chlorophenyl)-5H-dibenzo[b,e][1,4]-diazepine* (**27e**). Prepared from 4-chloro-2-fluoro-1-nitrobenzene and 2-amino-2’,4-dichloro-benzophenone (131 mg, 70% yield). ^1^H-NMR (600 MHz, CDCl_3_): δ/ppm = 7.54 (m, 1H), 7.40–7.36 (m, 3H), 7.18 (dd, *J =* 8.5 Hz, 2.4 Hz, 1H), 7.14 (d, *J =* 8.4 Hz, 1H), 6.98 (dd, *J =* 8.4 Hz, 2.2 Hz, 1H), 6.70 (d, *J =* 2.1 Hz, 1H), 6.65–6.63 (m, 2H), 5.47 (s, 1H); ^13^C-NMR (150 MHz, CDCl_3_): δ 167.7 (C), 151.5 (C), 143.6 (C), 140.0 (C), 138.4 (C), 133.6 (C), 132.6 (C), 132.2 (CH), 130.8 (CH), 130.6 (CH), 130.3 (CH), 130.2 (CH), 130.1 (CH), 129.7 (C), 128.4 (C), 127.0 (CH), 124.3 (CH), 120.9 (CH), 120.0 (CH); IR (neat) cm^−1^: 3257.3 (w, NH), 3051.1 (w), 2919.2 (w), 2850.0 (w), 1605.9 (m, N=C), 1489.2 (w), 1446.1 (m), 1434.2 (m), 1390.9 (w), 1315.9 (m), 1258.5 (w), 1236.7 (w), 1204.1 (w), 1155.6 (w), 1123.8 (w), 1085, 2 (w), 1057.6 (w), 968.8 (w), 942.8(w), 916.4(m), 880.9 (m), 857.7 (s), 822.1 (s), 747.5 (s), 727.9 (s); LC-MS: *t_r_* = 5.26, *m/z* = 374.94 (C_19_H_11_Cl_3_N_2_)^+^. The structure was unambiguously confirmed by X-ray crystallography. and deposited at the Cambridge Crystallographic Data Centre with the unique reference number CCDC 867822; Formula: C_19_H_11_Cl_3_N_2_, unit cell parameters: *a* = 15.5464(3) Å, *b* = 9.3738(2) Å, *c* = 24.0677(5) Å, *α* = 90°, *β* = 107.942(1)°, *γ* = 90°, space group: C2/*c*.

*8-Fluoro-11-phenyl-5H-dibenzo[b,e][1,4]diazepine* (**27f**). Prepared from 1,4-difluoro-2-nitrobenzene and 2-aminobenzophenone (192 mg, 67% yield). ^1^H-NMR (600 MHz, CDCl_3_): δ/ppm = 7.71 (d, *J =* 7.3 Hz, 2H), 7.48–7.40 (m, 3H), 7.32 (td, *J =* 7.6 Hz, 1.2 Hz, 1H), 7.04 (d, *J =* 9.2 Hz, 2H), 6.96 (t, *J =* 7.5 Hz, 1H), 6.79 (d, *J =* 7.9 Hz, 1H), 6.75 (td, *J =* 8.2 Hz, 2.8 Hz 1H), 6.66–6.63 (m, 1H), 4.95 (s, 1H); ^13^C-NMR (150 MHz, CDCl_3_): δ 170.6 (C), 159.8 (d, *J =* 240.7 Hz, C-F), 154.5 (C), 142.1 (d, *J =* 10.6 Hz, C), 140.9 (C), 138.7 (d, *J =* 2.5 Hz, C), 132.2 (d, *J =* 9.2 Hz, CH), 130.3 (CH), 129.7 (2 × CH), 128.0 (2 × CH), 127.5 (C), 122.6 (CH), 120.2 (d, *J =* 9.1 Hz, CH), 119.7 (2 × CH), 114.7 (d, *J =* 23.4 Hz, CH), 113.0 (d, *J =* 22.9 Hz, CH); IR (neat) cm^−1^: 3350.8 (w), 3061.9 (w), 1600.9 (m, N=C), 1570.4 (w), 1497.7 (m), 1461.6 (s), 1389.2 (w), 1318.2 (w), 1285.9 (w), 1257.3 (m), 1169.8 (w), 1135.0 (w), 1099.1 (w), 970.8 (m), 906.5 (w), 869.2 (w), 807.7 (m), 750.1 (s), 721.8 (s), 694.3 (s), 660.3 (m); LC-MS: *t_r_* = 4.99, *m/z* = 288.92 (C_19_H_13_FN_2_)^+^.

*2-Chloro-8-fluoro-11-phenyl-5H-dibenzo[b,e][1,4]diazepine* (**27g**). Prepared from 1,4-difluoro-2-nitrobenzene and 2-amino-4-chlorobenzophenone (81 mg, 50% yield) ^1^H-NMR (600 MHz, CDCl_3_): δ/ppm = 7.70 (d, *J =* 7.4 Hz, 2H), 7.50-7.42 (m, 3H), 7.28 (dd, *J =* 8.5 Hz, 2.3 Hz, 1H), 7.03 (dd, *J =* 9.5 Hz, 2.8 Hz, 1H), 7.00 (d, *J =* 2.3 Hz, 1H), 6.77–6.73 (m, 2H), 6.64 (m, 1H), 4.93 (s, 1H); ^13^C-NMR (150 MHz, CDCl_3_): δ 169.0, 159.9 (d, *J =* 241.4 Hz, C-F), 153.0, 141.8 (d, *J =* 10.8 Hz), 140.2, 138.2 (d, *J =* 2.6 Hz), 131.9, 131.6, 130.6, 129.5, 128.8, 128.3, 128.1, 121.1, 120.2 (d, *J =* 9.0 Hz), 114.8 (d, *J =* 23.5 Hz), 113.3 (d, *J =* 22.9 Hz); IR (neat) cm^−1^: 3272.0 (w, NH), 3064.0 (w), 1598.2 (w), 15.72.3 (w), 1491.6 (w), 1463.7 (s), 1318.2 (w), 1258.0 (s), 1206.4 (w), 1138.9 (w), 1119.2 (m), 1100.9 (w), 976.8 (w), 954.8 (w), 909.0 (w), 868.1 (m), 820.2 (m), 774.6 (w), 755.5 (m), 721.9 (s), 692.3(s); LC-MS: *t_r_* = 5.15, *m/z* = 322.84 (C_19_H_12_ClFN_2_)^+^.

*2,7-Dichloro-11-(2-fluorophenyl)-5H-dibenzo[b,e][1,4]diazepine* (**27h**). Prepared from 4-chloro-2-fluoro-1-nitrobenzene and 2-amino-4-chloro-2’-fluoro-benzophenone (105 mg, 33% yield). ^1^H-NMR (400 MHz, CDCl_3_): δ/ppm = 7.67 (td, *J =* 7.6 Hz, 1.9 Hz, 1H), 7.46–7.40 (m, 1H), 7.25 (dd, *J =* 7.5 Hz, 1.0 Hz, 1H), 7.22–7.19 (m, 1H), 7.15 (d, *J =* 8.5 Hz, 1H), 7.09–7.04 (m, 1H), 6.98 (dd, *J =* 8.6 Hz, 2.0 Hz, 1H), 6.83 (d, *J =* 2.4 Hz, 1H), 6.82–6.80 (m, 1H), 5.83 (s, 1H); ^13^C-NMR (100 MHz, CDCl_3_): δ 164.9 (C), 160.5 (d, *J =* 250.0 Hz, C-F), 151.1 (C), 143.6 (C), 138.7 (C), 133.2 (C), 132.1 (CH), 131.7 (d, *J =* 8.3 Hz, CH), 131.3 (d, *J =* 2.5 Hz, CH), 130.1 (CH), 130.1 (d, *J =* 1.7 Hz, C), 130.0 (C), 129.0 (d, *J =* 12.4 Hz, C), 128.4 (C), 124.3 (d, *J =* 3.3 Hz, CH), 124.2 (CH), 121.2 (CH), 120.0 (CH), 116.2 (d, *J =* 17.5 Hz, CH); IR (neat) cm^−1^: 3235.0 (w), 2949.9 (w), 2663.3 (w), 1610.7 (m), 1482.9 (m), 1454.4 (s), 1389.8 (w), 1316.2 (w), 1290.5 (w), 1237.0 (w), 1161.9 (w), 1089.1 (w), 1035.0 (w), 919.2 (w), 838.7 (w), 756.5 (w); HRMS (*m/z*) calculated for C_19_H_12_N_2_Cl_2_F (M+H) 357.0356; found 357.0349.

*13H-Benzo[b]fluoreno[1,9-ef][1,4]diazepine* (**27i**). Prepared from 1-fluoronitrobenzene and 2-aminofluorenone (120 mg, 51% yield). ^1^H-NMR (400 MHz, CDCl_3_): δ/ppm = 7.55 (d, *J =* 7.5 Hz, 1H), 7.28 (m, 2H), 7.20 (m, 1H), 6.85 (dd, *J =* 7.7 Hz, 1.5 Hz, 1H), 6.70 (t, *J =* 7.7 Hz, 1H), 6.66 (td, *J =* 7.7 Hz, 1.5 Hz, 1H), 6.45 (d, *J =* 7.4 Hz, 1H), 6.52 (dt, *J =* 7.4 Hz, 1.5 Hz, 1H), 5.82 (dd, *J =* 8.0 Hz, 1.5 Hz, 1H), 5.61 (d, *J =* 7.8 Hz, 1H), 4.41 (s, 1H, NH); ^13^C-NMR (100 MHz, CDCl_3_): δ 165.1 (C), 147.3 (C), 144.5 (C), 142.0 (C), 141.0 (C), 138.5 (C), 137.7 (C), 136.0 (CH), 135.4 (CH), 131.5 (CH), 130.9 (CH), 128.4 (CH), 123.3 (CH), 122.8 (CH), 121.8 (C), 120.2 (CH), 119.2 (CH), 114.3 (CH), 111.9 (CH). IR (neat) cm^−1^: 3400.1 (w), 3061.8 (w), 2925 (w), 1645.6 (w), 1623.4 (m), 1597.0 (m), 1466.0 (s), 1454.0 (m), 1434.5 (w), 1412.5 (w), 1321.8 (m), 1157.0 (w), 1058.7 (w), 958.5 (w), 779.1 (w), 747.5 (s). HRMS (*m/z*) calculated for C_19_H_13_N_2_ (M+H), 269.1073; found 269.1068.

*10-Fluoro-13H-benzo[b]fluoreno[1,9-ef][1,4]diazepine* (**27j**). Prepared from 1,4-difluoro-2-nitrobenzene and 2-aminofluorenone (105 mg, 37% yield). ^1^H-NMR (500 MHz, *d*_6_-DMSO): δ/ppm = 7.42 (dd, *J =* 7.2 Hz, 1.0 Hz, 1H), 7.37 (d, *J =* 1.0 Hz, 1H), 7.34 (m, 1H), 7.20 (td, *J =* 7.5 Hz, 1.0 Hz, 1H), 6.99 (m, 1H, NH), 6.70 (td, *J =* 7.5 Hz, 1.7 Hz, 1H), 6.53 (m, 1H), 6.46 (dd, *J =* 7.2 Hz, 0.7 Hz, 1H), 6.35 (dd, *J =* 9.8 Hz, 3.0 Hz, 1H), 6.02 (dd, *J =* 8.9 Hz, 5.8 Hz, 1H), 5.73 (dd, *J =* 7.5 Hz, 0.7 Hz, 1H); ^13^C-NMR (125 MHz, *d*_6_-DMSO): δ 166.0 (C), 157.6 (d, 295.2, C-F), 148.7 (C), 143.5 (C), 141.4 (C), 138.8 (d, *J =* 9.1 Hz, C), 138.7 (d, *J =* 3.3 Hz, C), 136.2 (CH), 136.0 (C), 131.9 (CH), 122.1 (CH), 120.8 (d, *J =* 28.4 Hz, CH), 120.4 (C), 120.4 (CH), 119.9 (d, *J =* 10.1 Hz, CH), 116.8 (d, *J =* 27.1 Hz, CH), 114.7 (C), 111.1 (CH). IR (neat) cm^−1^: 3390.0 (w, NH), 3060.0 (w), 2327.6 (w), 1650.0 (w), 1597.8 (s), 1468.1 (s), 1455.1 (m), 1421.7 (m), 1320.0 (w), 1266.3 (m), 1210.5 (w), 1160.3 (w), 974.7 (w), 957 (w), 878.5 (w), 749.4 (m). HRMS (*m/z*) calculated for C_19_H_12_N_2_F (M+H), 287.0979; found 287.0972.

*11-Chloro-13H-benzo[b]fluoreno[1,9-ef][1,4]diazepine* (**27k**). Prepared from 4-chloro-2-fluoro-1-nitrobenzene and 2-aminofluorenone (105 mg, 42% yield). ^1^H-NMR (500 MHz, *d*_6_-DMSO): δ/ppm = 7.43 (dd, *J =* 7.1 Hz, 1.0 Hz, 1H), 7.37 (d, *J =* 1.4 Hz, 1H), 7.34 (dd, *J =* 7.8 Hz, 1.0 Hz, 1H), 7.21 (td, *J =* 7.5 Hz, 1.0 Hz, 1H), 7.18 (m, 1H, NH), 6.73 (t, *J =* 8.0 Hz, 1H), 6.56 (d, *J =* 8.1 Hz, 1H), 6.52 (d, *J =* 7.5 Hz, 1H), 6.41 (dd, *J =* 8.5 Hz, 2.4 Hz, 1H), 6.09 (d, *J =* 2.4 Hz, 1H), 5.73 (dd, *J =* 8.2 Hz, 0.7 Hz, 1H); ^13^C-NMR (125 MHz, *d*_6_-DMSO): δ 164.2 (C), 147.4 (C), 143.4 (C), 1471.3 (C), 136.6 (CH), 136.5 (C), 136.0 (C), 135.9 (CH), 134.8 (C), 131.8 (CH), 128.3 (CH), 122.0 (CH), 121.4 (CH), 120.8 (C), 120.5 (CH), 118.2 (CH), 114.7 (CH), 111.81 (CH). IR (neat) cm^−1^: 3395.1 (w, NH), 2926.3 (m), 2851.7 (m), 2308.1 (w), 1720.5 (w), 1650.0 (w), 1627.2 (w), 1598.5 (m), 1575.6 (m), 1464.8 (s), 1422.2 (m), 1314.2 (w), 1104.5 (w), 966.6 (w), 806.7 (w), 778.7 (w), 750.4 (m). HRMS (*m/z*) calculated for C_19_H_12_N_2_Cl (M+H), 303.0684; found 303.0675. 

Crystallographic data for compounds **27b**, **27d** and **27e** have been deposited with the accession numbers CCDC 867822, 867823 and 867824contains the supplementary crystallographic data for this paper. These data can be obtained free of charge via http://www.ccdc.cam.ac.uk/conts/retrieving.html (or from the CCDC, 12 Union Road, Cambridge CB2 1EZ, UK; Fax: +44 1223 336033; E-mail: deposit@ccdc.cam.ac.uk)

## 4. Conclusions

In summary, we have successfully extended on our earlier reports detailing the flow-based chemoselective reduction of important nitro substituted building blocks. This efficient methodology allowed the rapid generation of valuable aminobenzene intermediates, which were used as starting points in accessing important heterocyclic scaffolds such as riboflavines, quinoxalinones and dibenzodiazepines. Importantly, the mild reaction conditions of the developed protocols allow for directly employing substrates bearing valuable yet sensible halide functionalities. 
